# Successful balloon valvuloplasty of a subpulmonic membrane associated with cor triatriatum dexter: a case report

**DOI:** 10.1186/s13256-019-2218-1

**Published:** 2019-09-15

**Authors:** Meryem Haboub, Abdenasser Drighil

**Affiliations:** Cardiology Department, Hospital University Ibn Rochd, Casablanca, Morocco

**Keywords:** Subpulmonic membrane, Cor triatriatum dexter, Balloon valvuloplasty

## Abstract

**Background:**

Subpulmonic membrane as a cause of right ventricular outflow tract obstruction in patients with concordant ventriculoarterial connection and intact ventricular septum is considered to be rare. Association with cor triatriatum dexter and success of subpulmonic balloon valvuloplasty have never been reported, at least to the best of our knowledge.

**Case presentation:**

A 3-year-old Moroccan boy was referred to our tertiary care hospital with complaints of dyspnea on moderate exertion. A physical examination revealed parasternal lift, systolic thrill, and a 4/6 ejection systolic murmur, best heard over the left second intercostal space. His oxygen saturation was 99% on room air. Two-dimensional echocardiography showed a discrete circumferential membrane just below the pulmonic valve and a right atrial membrane. Continuous wave Doppler interrogation showed peak systolic pressure gradient of 85 mmHg across the subpulmonic membrane and no significant gradient across the right atrial membrane. Balloon dilation of the subpulmonic membrane was performed and the pressure gradient came down to 50 mmHg. During follow-up, he reported marked improvement in terms of exercise tolerance. Transthoracic echocardiography showed residual pressure gradient of approximately 40 mmHg across the membrane. Surgery resection of the two membranes was programmed, but he died after an extracardiac disease (appendicular peritonitis).

**Conclusions:**

Subpulmonic membrane as an isolated cause of right ventricular outflow tract obstruction is rare. Its association with cor triatriatum dexter is even less common. The result of percutaneous balloon valvuloplasty of subpulmonic membrane is an interesting alternative while waiting for surgery. Surgery is currently the preferred modality of treatment with the resection of both right atrial and subpulmonic membranes.

## Background

Congenital heart diseases producing obstruction of the right ventricular outflow tract (RVOT) are relatively common and include abnormalities at the mid-right ventricle, the infundibulum, the pulmonary valve, and supravalvular or the branch and/or peripheral pulmonary arteries. Subvalvular pulmonary stenosis commonly occurs as muscular hypertrophy associated with Tetralogy of Fallot or ventricular septal defect (VSD) [1].

Membranous subpulmonary stenosis is rare, and only a few cases have been reported, mostly in association with other congenital defects like aortic regurgitation and VSD [2], supracristal VSD [3], severe pulmonary valvar stenosis [4], and congenitally corrected transposition of the great vessels, and, in this particular case, balloon valvuloplasty of the subpulmonic membrane was successful [5]. Isolated subpulmonary membranes are extremely rare and only two cases have been reported in the literature [6, 7].

Cor triatriatum dexter, or partitioning of the right atrium (RA) to form a triatrial heart, is an extremely rare congenital anomaly that is caused by the persistence of the right valve of the sinus venosus [8]. The incidence of cor triatriatum is approximately 0.1% of congenital heart malformations [9]. Typically, the right atrial partition is due to exaggerated fetal Eustachian and Thebesian valves, which together form an incomplete septum across the lower part of the atrium. This septum may range from a reticulum to a substantial sheet of tissue [8]. Cor triatriatum dexter can occur as an isolated cardiac anomaly or be associated with other malformations of structures of the right side of the heart, such as pulmonary artery stenosis or atresia, atrial septal defect, or Ebstein anomaly [10, 11].

This case is presented because it is, at least to the best of our knowledge, the first case report to describe cor triatriatum dexter associated with an obstructive subpulmonic membrane and a successful attempt of balloon valvuloplasty of the subpulmonic membrane.

## Case presentation

### Patient information

We describe a 3-year-old Moroccan boy with no past medical, social, environmental, or family history who was referred to our department with dyspnea on moderate exertion for 3 months. His parents were not related. He was not on any medication at the time of presentation.

### Clinical findings

On physical examination, he appeared to be a well-grown and healthy appearing boy with a weight of 15 kg, height of 80 cm, and body mass index of 23.4 kg/m^2^. His blood pressure was 100/60 mmHg and his pulse rate was 100 beats per minute (bpm). His oxygen saturation was 99% on room air. Peripheral pulses were palpable. He had a parasternal lift, systolic thrill, and a 4/6 ejection systolic murmur, best heard over the left second intercostal space, and a pansystolic murmur of grade 3/6 intensity, best heard over the left lower sternal border. There was no hepatomegaly or peripheral edema. A neurological examination and a general physical examination were normal.

### Diagnostic assessment

**Fig. 1:**
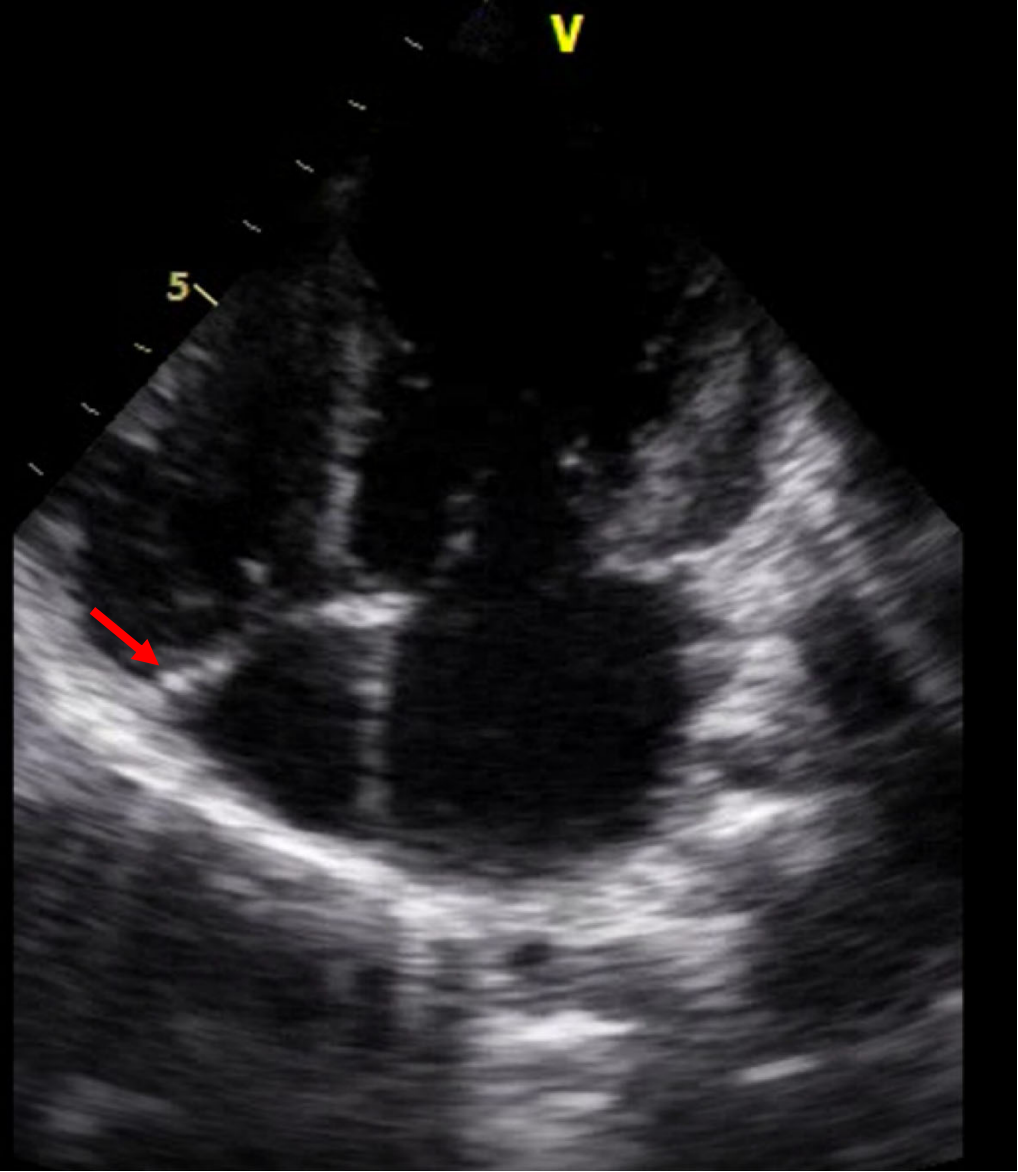
Apical four-chambers view of a transthoracic echocardiogram showing the membrane partitioning the right atrium (arrow)

**Fig. 2:**
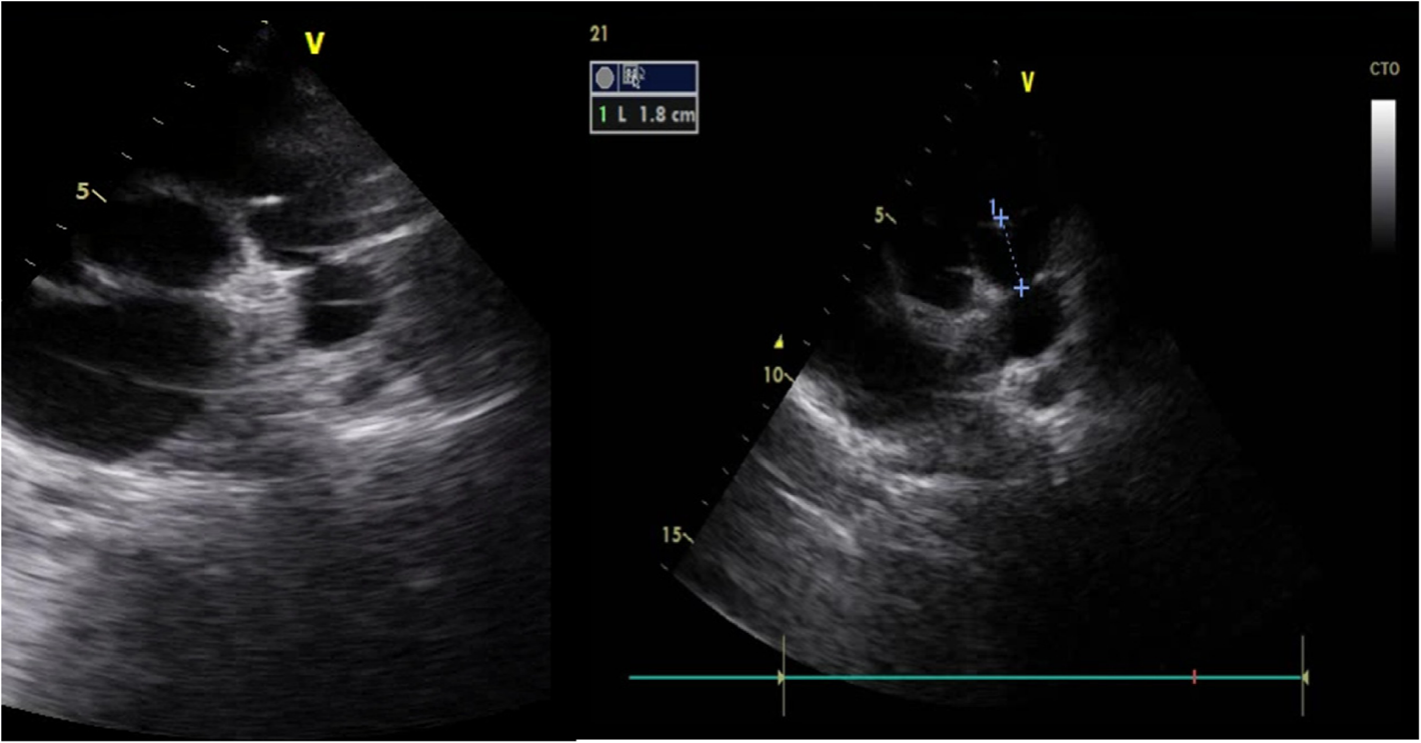
Parasternal short-axis view of a transthoracic echocardiogram showing subpulmonic membrane standing 18mm below pulmonic valve leaflets

**Fig. 3:**
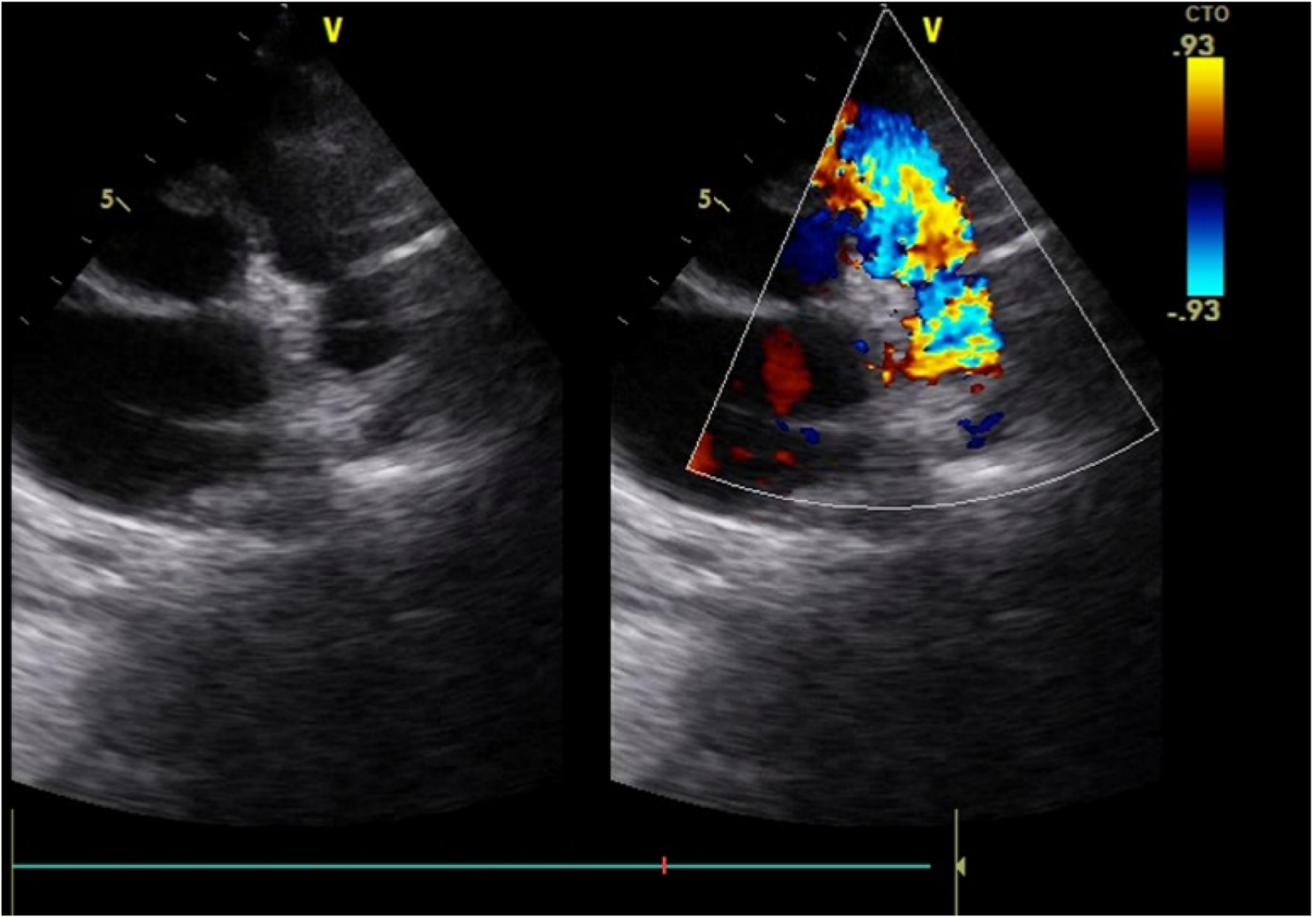
Parasternal short-axis view of a transthoracic echocardiogram with color Doppler showing an aliasing through the subpulmonic membrane

His chest X-ray showed a cardiothoracic index at 0.55 with clearly decreased pulmonary vascular markings. An electrocardiogram showed a sinus rhythm with a rate of 100 bpm, no axis deviation, right atrial enlargement, negative T waves in DI and aVL, and normal PR and QT intervals. An echocardiographic examination showed normal abdominal and atrial situs, normal position of the heart in the left side of his chest, and normal venoatrial, atrioventricular, and ventriculoarterial relationships. The right ventricle looked markedly dilated and hypertrophied, and right ventricular systolic function appeared grossly reduced on visual inspection and tricuspid annular plane systolic excursion (TAPSE). The left ventricle was not dilated or hypertrophic; its systolic function was preserved. The mitral and aortic valves were structurally normal. The interventricular and interatrial septum appeared intact. The RA was dilated with a membrane partitioning it largely perforated at 18 mm without gradient through it at continuous wave Doppler interrogation (Fig. 1). The tricuspid annulus was hypoplastic (diameter at 14 mm) with a 5 mmHg of mean gradient on continuous wave Doppler interrogation. There was a discrete circumferential membrane 18 mm below the pulmonic valve and continuous wave Doppler interrogation across the membrane showed a peak systolic pressure gradient at 85 mmHg (Figs. 2 and 3). The pulmonic valve was thin, had normal pressure gradients, and pulmonary annulus at 14 mm. Right and left pulmonary arteries were confluent and good sized. Coronary sinus was dilated without a seen left superior vena cava or abnormal pulmonary venous return or a stenosis of its foramen. The left aortic arch had no coarctation and no patent ductus arteriosus. There was no pericardial effusion. His inferior vena cava was dilated at 16 mm.

A complete blood count showed hemoglobin at 13 g/dl, white blood cells at 7100 cells/mcl, and platelets at 310,000 cells/mcl. Liver function tests were normal with an alanine aminotransferase (ALT) of 17 IU/L and an aspartate aminotransferase (AST) of 21 IU/L. Renal function tests were also normal with a serum creatinine level at 0.5 mg/dl and a blood urea nitrogen level at 7 mg/dl.

### Therapeutic intervention, follow-up, and outcomes

**Fig. 4:**
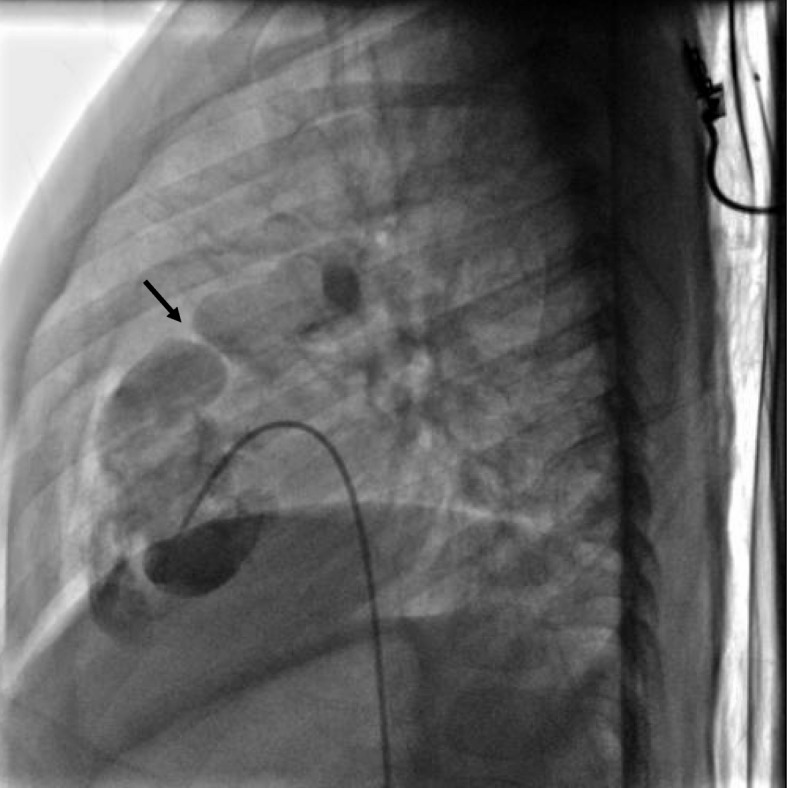
Ventriculogram of the right ventricle showing the subpulmonic membrane (*arrow*)

Balloon valvuloplasty of subpulmonic membrane was performed, using Tyshak II® balloon PDC501 5.0 mm, leading to symptoms improvement and regression of maximal gradient through the membrane from 85 to 50 mmHg (Fig. 4). No medication was used. Surgical resection of right atrial and subpulmonic membranes was planned but our patient died 1 year after the intervention because of an extracardiac disease (appendicular peritonitis). An autopsy was not performed.

## Discussion

Association of cor triatriatum dexter and subpulmonic membrane with a successful attempt of balloon valvuloplasty is very rare and this case is, at least to the best of our knowledge, the first one to be reported in the literature.

During embryogenesis, the right horn of the sinus venosus is gradually incorporated into the RA to form the smooth posterior portion of the RA, whereas the original embryologic RA forms the trabeculated anterior portion. The connection between the right horn of the sinus venosus and the embryologic RA is the sinoatrial orifice, which is flanked on either side by two valvular folds, the right and left venous valves. At some point during this incorporation, the right valve of the right horn of the sinus venosus divides the RA into two. This right valve forms a sheet that serves to direct the oxygenated venous return from the inferior vena cava across the foramen ovale to the left side of the heart during fetal life [8]. Normally, the valve regresses by approximately 12 weeks of gestation and leaves behind the crista terminalis superiorly and the Eustachian valve of the inferior vena cava and the Thebesian valve of the coronary sinus inferiorly. Complete persistence of the right sinus valve results in a separation between the smooth and trabeculated portions of the RA, constituting cor triatriatum dexter [10].

Cor triatriatum dexter can occur as an isolated cardiac anomaly or be associated with other malformations of structures of the right side of the heart, such as pulmonary artery stenosis or atresia, atrial septal defect, or Ebstein anomaly [10, 11].

Unlike cor triatriatum sinister, which carries a high mortality rate if not repaired, cor triatriatum dexter has varying clinical manifestations depending on the degree of partitioning or septation of the RA. When the septation is mild, the condition is often asymptomatic and is an incidental finding during surgery to correct other cardiac abnormalities or during echocardiography. In our case, the septation of the RA was mild with a large orifice of 18 mm and no significant gradient through it, the symptomatology was essentially due to severe RVOT obstruction with a maximal gradient through the subpulmonic membrane at 85 mmHg.

Asymptomatic patients with cor triatriatum dexter are generally not treated unless they are undergoing cardiac surgery for other reasons. In the past, the mainstay of treatment for symptomatic patients has been surgical resection of the dividing membrane [9, 11]. Recently, percutaneous catheter disruption of the membrane has been reported for obstructive right atrial membranes and has been suggested as a preferred alternative to open heart surgery [12].

RVOT obstructions are relatively common and subpulmonic obstructions are usually due to infundibular hypertrophy. Other causes of subpulmonic obstruction reported in the literature include aneurysm of the membranous septum [5, 6], aneurysmal tricuspid valve tissue in the setting of membranous VSD [7], double-chambered right ventricle [13], and unruptured sinus of Valsalva aneurysm [14, 15]. A single case of RVOT obstruction resulting from a tuberculoma in a patient with VSD and aneurysm of the membranous septum has also been reported [16]. Subpulmonic membrane as a cause of RVOT obstruction is rare, and only a few cases have been reported, mostly in association with other congenital defects like aortic regurgitation and VSD [2], supracristal VSD [3], severe pulmonary valvar stenosis [4], and congenitally corrected transposition of the great vessels, and, in this particular case, balloon valvuloplasty of the subpulmonic membrane was successful [5]. Isolated subpulmonary membranes are rare and only two cases have been reported in the literature [6, 7]. Our case will be the first reported one associating cor triatriatum dexter and a subpulmonic membrane.

There is no embryologic link between subpulmonic membrane and cor triatriatum dexter.

Balloon valvuloplasty has proven clinical benefits in several settings. In adults, this technique when applied to the aortic valve provides effective short-term palliation in patients who are not optimal surgical candidates. In some cases, it is the procedure of choice for patients with mitral stenosis not complicated by left atrial thrombi, significant mitral regurgitation, or marked thickening of the subvalvular apparatus. It has also become the technique of choice for relief of pulmonic stenosis, particularly in the pediatric population. Balloon dilatation procedures are also increasingly applied to coarctation of the aorta. Although the efficacy of balloon dilation of subaortic membranes has been reported, successful dilation of a subpulmonic membrane has been reported in only one case, a 22-year-old woman with a congenitally corrected transposition of the great vessels and an obstructive subpulmonic membrane [5]. In our case, balloon valvuloplasty of the subpulmonic membrane was also successful, leading to symptoms improvement and regression of the transmembrane maximal gradient from 85 to 50 mmHg at follow-up echocardiogram. His post valvuloplasty echocardiogram suggested that the residual gradient was mainly due to subvalvar component. Although there is a report of success in dilating the membrane itself [5], it is understandable that balloon dilation of the valve is less likely to result in complete elimination of the RVOT gradient. Surgery is the currently preferred modality of treatment, which can result in complete resolution of the RVOT obstruction [6]. When the subpulmonic membrane is too close to the pulmonic valve one needs to be cautious, as there is a risk of damage to the valve leaflets during surgery [6].

In our case, the obstructive subpulmonic membrane was successfully dilated by percutaneous balloon valvuloplasty and no percutaneous intervention was taken to treat the partitioning membrane in the RA. Surgical resection of the subpulmonic membrane and RA partitioning membrane was programmed, but our patient died in the context of appendicular peritonitis before it could be done.

## Conclusions

Subpulmonic membrane as an isolated cause of RVOT obstruction is rare. Its association with cor triatriatum dexter is even less common. The result of percutaneous balloon valvuloplasty of subpulmonic membrane is an interesting alternative while waiting for surgery. Surgery is currently the preferred modality of treatment with the resection of both right atrial and subpulmonic membranes.

## Data Availability

The published information is available from the corresponding author on reasonable request.
